# Relationship of segment area and monitor unit efficiency in aperture‐based IMRT optimization

**DOI:** 10.1120/jacmp.v14i3.4056

**Published:** 2013-05-06

**Authors:** Peng Qi, Ping Xia

**Affiliations:** ^1^ Department of Radiation Oncology Cleveland Clinic Cleveland OH USA

**Keywords:** monitor units, intensity‐modulated radiotherapy, optimization, prostate cancer, nasopharyngeal cancer

## Abstract

In step‐and‐shoot IMRT plans, aperture‐based optimization (or one‐step optimization) has been considered as a means of improving monitor unit (MU) efficiency compared to fluence‐based optimization (or two‐step optimization). However, the extent of improvement on MU efficiency varies, depending on the implementation and design of one‐step optimization. In this paper, we attempted to investigate MU efficiency issue in two methods of one‐step optimization implemented in two commercial treatment planning systems (TPSs). Five patients with nasopharyngeal cancer and five patients with advanced prostate cancer were selected for this study. For these patients, clinically used IMRT plans were generated using the Direct Machine Parameter Optimization (DMPO) in the Pinnacle TPS. New IMRT plans were created using the Direct Aperture Optimization (DAO) method in the Panther TPS. For the purpose of this study, we used the similar planning dose objectives and beam configurations with a similar total number of segments in each pair of DMPO and DAO plans. With similar plan quality, DMPO plans required more MUs than DAO plans. The average number of MUs (expressed in mean ±1 SD) for the DMPO and DAO plans was 1,169±186 and 671±135 for the nasopharynx cases, and 711±48 and 400±65 for the prostate cases, respectively. The average segment areas (expressed in mean ±1 SD) for the DMPO plans were smaller than those for the DAO plans: $46.0\pm 7.6\;{\text{cm}}^{2}$ vs. $100.9\pm 32.3\;{\text{cm}}^{2}$ for the nasopharynx cases, and $58.3\pm 17.2\;{\text{cm}}^{2}$ vs. $97.4\pm 35.0\;{\text{cm}}^{2}$ for the prostate cases, respectively. In conclusion, two one‐step optimization algorithms, DMPO and DAO, resulted in much different MU efficiency with the similar number of segments and optimization parameters. This MU difference is largely attributed to the fact that large area segments are used more often in DAO plans than in DMPO plans.

PACS number: 87.55.de

## I. INTRODUCTION

Intensity‐modulated radiotherapy (IMRT) can achieve conformal dose distributions to concave tumor shape while sparing nearby normal tissues. Step‐and‐shoot IMRT plans can be created using a fluence‐based optimization involving two steps: a fluence optimization and a delivery optimization (or a leaf‐sequencing process). This type of optimization is referred to as two‐step optimization.^(1)^ In order to faithfully deliver the optimized fluence, the leaf sequencer often produces many small area segments (or control points), resulting in increased monitor units (MUs) compared with those in 3D conformal radiotherapy (3D CRT). This MU increase has caused some concerns regarding elevation of secondary cancer,^2^, ^3^ the need for more stringent requirement in room shielding, and protracted treatment time. To overcome this problem, various aperture‐based optimization algorithms, referred to as one‐step optimization, were proposed to eliminate the step of fluence optimization in two‐step optimization algorithms.^4^, ^5^, ^6^, ^7^, ^8^, ^9^, ^10^, ^11^, ^12^ There is renewed interest in rotational radiotherapy techniques to achieve improvement of MU efficiency and treatment throughput for IMRT delivery.^(13)^

In their early study of direct aperture optimization (DAO), Shepard et al.^(5)^ showed that the number of segments and the number of MUs for a DAO plan of a head‐and‐neck cancer were 85% and 74% fewer than those for an IMRT plan created with a two‐step optimization method. Bergman et al.^(10)^ introduced a Monte Carlo‐based DAO algorithm. For a nasopharynx case, they found approximately 33% improvement in MU efficiency when the optimization engine was changed from two‐step optimization to one‐step optimization. Many commercial treatment planning systems (TPSs) now offer one‐step optimization for step‐and‐shoot IMRT plans.

Recently, several studies reported clinical comparisons of one‐step and two‐step optimization in the Pinnacle TPS. In this TPS, the one‐step optimization is referred to as the Direct Machine Parameter Optimization (DMPO), and the two‐step method is referred to as the Intensity Modulation (IM). From a study of 11 head‐and‐neck plans, Jones and Williams^(13)^ found that 42% fewer MUs and 35% fewer segments were used in DMPO plans than corresponding IM plans. Ludlum and Xia^(1)^ showed that a 10%–15% MU reduction was achieved when changing from IM to DMPO for five prostate cases, but no significant MU reduction for five nasopharynx cases. DMPO and IM IMRT plans were also compared by van Asselen et al.^(14)^ for twelve breast cancer patients. They found no significant reduction in MUs, but a reduction in the number of segments. As well, Ahunbay et al.^(15)^ compared the DAO method in the Panther TPS and the two‐step optimization in the XiO CMS TPS for ten cases of whole breast treatment. They observed that the total number of MUs for DAO plans were approximately 60% less than those of two‐step optimization IMRT plans.

As observed from these published studies,^1^, ^5^, ^10^, ^14^, ^15^ one‐step optimization improves MU efficiency when compared to the two‐step optimization. However, the extent of MU reduction varied substantially due to differences in the design and/or implementation among one‐step optimization algorithms, as well as the complexity of disease sites. Instead of comparing one‐and two‐step optimization as in those previous studies, we studied two one‐step optimization methods implemented in two commercial TPSs. Two common IMRT treatment sites, prostate and head and neck, were used to evaluate the effect of different plan complexity. While keep the similar number of segments and optimization parameters, we attempted to understand what other factors may impact MU efficiency for step‐and‐shoot IMRT plans created with one‐step optimization algorithms.

## II. MATERIALS AND METHODS

### A. Two treatment planning systems

For this study, clinical IMRT plans were generated using DMPO in the Pinnacle (version 8.0m; Philips, Andover, MA) TPS and DAO in the Panther (version 4.6; Prowess, Concord, CA) TPS. In the process of DMPO, the first n iterations (locally set to about 30% of the total iterations N) are used for fluence‐based optimization. After the nth iteration, a leaf sequencer converts the semioptimized fluence map to a preset number of deliverable MLC segments while considering machine‐specific delivery parameters and user‐defined parameters, such as the minimum segment area (MSA) per segment and minimum number of MUs per segment. These converted MLC segments are then used as a starting point for the subsequent aperture‐based optimization in the remaining (N‐n) iterations. During these iterations, leaf positions and segment weights (which directly associate with MUs per segment) are iteratively adjusted to minimize the value of a cost function. The Pinnacle TPS uses a gradient search method for its DMPO, which accepts every variable change that decreases the value of the cost function and discards any variable change that increases the value of the cost function.

In the Panther TPS, the planner selects beam energies, angles, and the number of segments per beam angle, but does not define the number of iterations. As for DMPO, the planner can preset the MSA and the minimum number of MUs per segment. With the DAO method, the shape of each aperture (or segment) at the beginning of the optimization is conformal to the projected target volume in the beam's eye view (BEV) of each beam with an equal beam weighting. During the optimization, the leaf positions and MUs (weighting) of each segment are adjusted simultaneously while minimizing the value of a cost function. A simulated annealing method is used as the iterative method for DAO. This stochastic method can accept a variable change that increases the value of the cost function at a certain probability. Theoretically, the simulated annealing approach is more likely to find the globe minimum than the gradient search method, but it does require longer optimization time with more iterations. Although DAO has been used as a generic term for one‐step optimization,^(16)^ DAO in this paper specifically refers to the optimization method implemented in the Panther TPS, which was based upon the algorithm proposed by Shepard et al.^(5)^

### B. Patient selections and plan objectives

Five patients with nasopharyngeal cancer and five patients with advanced prostate cancer, who underwent IMRT treatment with DMPO plans, were randomly selected for this study. The planning goals for patients with nasopharyngeal cancer were to treat 95% of the planning tumor volume (PTV70) and 100% of the clinical tumor volume (CTV70) to 70 Gy and to treat 95% of other PTVs and 100% of corresponding CTVs to the prescribed doses. For three patients, 59.4 Gy (1.8 Gy per fraction for 33 fraction) was prescribed to treat the PTV59.4. For two other patients, 63.0 Gy and 57.0 Gy were prescribed to treat the PTV63 and PTV57, respectively. The tolerance doses to critical structures, such as the brain stem, spinal cord, mandible, temporal lobe, parotid glands, inner ears, and larynx, were planned to meet the requirements of the RTOG HN‐0225 protocol.^(17)^

For patients with advanced prostate cancer, the treatment protocol consisted of two sequential plans: initial IMRT + boost IMRT or initial IMRT + boost brachytherapy. In this study, we only compared the initial IMRT plans. The treatment goals were to deliver a prescription dose of 45 Gy to 95% of the PTV and 100% of the pelvic lymph nodes, and to concurrently treat 95% of the PTV and 100% of the prostate and seminal vesicle to a prescribed dose (from 45 to 54 Gy) over 25 fractions. For critical structures, the plan acceptance criteria included that doses to 5% of the bladder and rectum volumes (D5) were less than the prescription dose of the prostate, while minimizing the volume of the small bowel to receive a dose greater than 45 Gy.

The typical planning dose constraints for both nasopharyngeal cancer and advanced prostate cancer are described elsewhere.^18^, ^19^ For both groups of patients treated with IMRT, we normalized the plan to the point of the maximum dose.

### C. DMPO and DAO plans

For each nasopharyngeal cancer patient, the clinical IMRT plan was generated using a beam configuration of eight to nine nonequally spaced coplanar beams. The intensity modulation for each 6 MV beam was optimized using the DMPO algorithm with the following optimization parameter settings: $\text{MSA}=2\;{\text{cm}}^{2}$, minimum monitor units =2 MU,n=10,N=25−30, and approximately 60–65 total number of segments (TNS). For each prostate cancer patient, the IMRT plan was produced with seven coplanar beams of 18 MV photon energy. The DMPO settings for the prostate cases were similar to those for the nasopharynx cases except for fewer segments (about 45). The settings of the number of segments were close to minimum numbers based upon recommendations of previous studies.^1^, ^20^

To compare with DMPO plans, we retrospectively created corresponding DAO plans in the Panther TPS. For each case, we transferred the planning CT images and contours from the Pinnacle TPS to the Panther TPS via a DICOM format. Beam configurations, such as beam angles, locations of isocenters, and energies in the DAO plans were kept the same as those in the corresponding DMPO plans, except collimator angles. For DMPO plans, the collimator was set at 0° collimator angle. For DAO plans, the use of 90° collimator angle often had improved plan quality than those using of 0° collimator angle. This plan quality dependence on collimator angles may arise from the design of the Prowess planning system and its discussion is beyond the scope of this study. On the other hand, we have compared DMPO plans created with 0° and 90° collimator angles for a variety of cancer sites, including the prostate and nasopharynx. Based upon our experience, plan quality and delivery efficiency were very similar for those DMPO plans. Therefore, it is a fair, though not an ideal, comparison between DMPO plans created with 0° collimator angles and DAO plans created with 90° collimator angles. Same as for DMPO plans, the MSA and minimum MUs of any deliverable segment was set as 2 cm^2^ and 2 MU for DAO plans, respectively. The number of segments per beam was set to ensure the total number of segments for a DAO plan comparable to that for the corresponding DMPO plan.

All IMRT plans were designed for delivery on a Siemens ONCOR linear accelerator (Siemens, Malvern, PA) equipped with an 82‐leaf MLC, with 1 cm leaf width projected at the isocenter.

### D. Plan comparison and evaluation

The Pinnacle TPS adapts a convolution/superposition algorithm based on the work of Mackie et al.^(21)^ and Papanikolaou et al.^(22)^ For the clinical plans, the adaptive convolution algorithm was used for the final dose calculation with a 4 mm dose grid resolution. The Panther system uses a collapsed cone convolution algorithm for its final dose calculation with a dose grid resolution of 3 mm, which is the only option for this research version of the Panther system. Because we preferred to use the original plan as our standard, these plans were not recalculated using a smaller dose resolution.

For each patient, the DMPO and DAO plan were compared based upon the number of segments, the number of MUs, tumor coverage, defined endpoints for sensitive structures, dose conformality indices (COINs) of target volumes, and a homogeneity index (HI). The conformality index and homogeneity index followed the RTOG report,^(23)^ which was listed in the review article by Feuvret et al.^(24)^ The target COIN was then defined as follows:

(1) $$\text{COIN}=V_{\text{RI}}/\text{TV}$$

where $V_{\text{RI}}$ is the volume of the reference isodose (here is the prescription isodose) and TV is the target volume. Ideally, COIN=1. The homogeneity index (HI) was defined as the ratio of the maximum dose ($D_{\text{max}}$) of the plan and the prescription dose (Rx),

(2) $$\text{HI}=D_{\text{max}}/\text{Rx}$$

### E. Analysis of segment information

For each IMRT plan, distributions of segment areas (SAs) and MUs per segment were calculated by using an in‐house program. Analysis of these distributions was performed by using Excel software's built‐in function, FREQUENCY. The bin size for the analysis of SAs was 10 cm^2^, except for the first bin element due to 2 cm^2^ MSA setting. For the analysis of segment MUs, a bin size of 3 MU was used, except that 2 MU was used as the lower limit of the first bin. The definition of SA in this work was the summation of every rectangle formed by any opening pair of MLC leaves.

### F. Effects of DMPO optimization parameters on MU efficiency

Unlike DAO plans, DMPO plans had many segments with sizes approaching the MSA setting of 2 cm^2^. To further investigate impacts of the MSA setting on the plan quality, we created new DMPO plans by using the same optimization parameters but with incrementally increased MSA values (such as 4, 8, 12, and 16 cm^2^). After resetting beams, a newly optimized DMPO plan was created and compared with the clinically approved plan based upon the cumulative dose‐volume histograms (DVHs) of the tumor target and critical structures. If DVHs in the new and clinical plans deviated and violated the original treatment guidelines, the process was terminated.

## III. RESULTS

### A. Plan evaluation and comparison

For the nasopharynx cases, the selected endpoint doses to the target volumes and organs at risk (OARs), HIs, number of segments, and number of MUs of DMPO and DAO plans are listed in Table 1. As seen in Table 1, the number of MUs was the only parameter with considerable differences between the DMPO and DAO plans. The mean MUs (expressed in mean ±1 SD) for five DMPO and DAO plans were 1,169±186 and 671±135, respectively.

Table 2 summarizes the detailed comparisons of the DMPO and the DAO plans for five patients with advanced prostate cancer. Similar to the nasopharynx cases, DMPO and DAO achieved similar treatment goals, but resulted in the mean MUs (expressed in mean ±1 SD) of 711±48 and 400±65, respectively. The prostate COINs were large due to fact that (1) the prescription dose to the nodes and prostate were not much different, and (2) the prostate is small compared to the pelvic lymph nodes.

**Table 1:** Plan evaluation and comparison for five patients (A–E) with nasopharyngeal cancer.

	*Plan Information*					
*Item (unit)*	*Type*	*A*	*B*	*C*	*D*	*E*
No. of monitor units	DMPO	1,160	963	1,330	1,381	1,011
	DAO	510	774	699	820	554
No. of segments	DMPO	54	67	58	60	69
	DAO	59	65	58	65	56
Ave. segment area (cm^2^)	DMPO	46.4	47.7	37.6	40.8	57.4
	DAO	136.7	63.1	83.3	88.8	132.8
	*Dosimetric Information*					
	*Type*	*A*	*B*	*C*	*D*	*E*
D95 of CTV70 (Gy)	DMPO	70.4	71.3	69.7	69.4	71.6
	DAO	70.5	71.3	70.0	70.7	71.4
D95 of CTV63^a^ or CTV_59.4^b^	DMPO	60.0^b^	59.5^b^	67.0^a^	64.4^a^	58.8^b^
	DAO	59.9^b^	59.5^b^	66.9^a^	64.3^a^	58.4^b^
$D_{\max}$ of spinal cord (Gy)	DMPO	45.0	43.2	43.5	43.7	45.8
	DAO	44.4	43.0	42.1	40.7	44.7
$D_{\max}$ of brain stem (Gy)	DMPO	55.2	56.7	53.6	53.9	55.2
	DAO	52.5	53.8	54.9	53.7	48.7
$D_{\mathrm{mean}}$ of parotidL (Gy)	DMPO	24.9	27.7	24.1	22.4	24.9
	DAO	25.4	25.4	25.8	25.8	30.2
$D_{\mathrm{mean}}$ of parotidR (Gy)	DMPO	28.1	25.8	25.7	23.5	26.4
	DAO	28.2	25.8	27.0	26.4	25.8
HI	DMPO	1.16	1.20	1.14	1.14	1.22
	DAO	1.19	1.14	1.23	1.17	1.18
Volume of CTV70 (cc)		49.1	81.9	225.0	115.7	106.6
Volume of CTV (cc)		818	496.0	300.0	257.3	729.2
COIN of CTV70	DMPO	5.78	3.69	1.29	1.34	2.49
	DAO	5.54	2.46	1.42	1.45	2.89
COIN of CTV	DMPO	1.53^b^	1.67^b^	1.71^a^	1.80^a^	1.33^b^
	DAO	1.58^b^	1.35^b^	1.96^a^	2.60^a^	1.31^b^

^a^ CTV63

^b^ CTV59.4

**Table 2:** Plan evaluation and comparison for five patients (F–J) with prostate cancer.

	*Plan Information*					
*Item (unit)*	*Type*	*F*	*G*	*H*	*I*	*J*
No. of monitor units	DMPO	764	719	650	675	746
	DAO	458	389	341	336	475
No. of segments	DMPO	45	50	45	40	39
	DAO	42	49	45	43	39
Ave. segment area (cm^2^)	DMPO	39.8	85.3	50.8	63.1	52.5
	DAO	60.2	146.6	88.7	118.2	73.4
	*Dosimetric Information*					
	*Type*	*F*	*G*	*H*	*I*	*J*
D95 of prostate (Gy)	DMPO	49.9	50.5	47.2	45.6	47.7
	DAO	50.4	50.3	46.3	46.0	47.6
D95 of nodes (Gy)	DMPO	45.5	44.8	46.0	43.7	46.0
	DAO	45.5	44.7	45.3	40.7	45.3
HI	DMPO	86.0	88.0	83.0	85.0	83.8
	DAO	85.6	90.0	86.5	83.5	84.4
Volume of prostate (cc)		35.1	17.0	40.0	63.1	36.0
Volume of nodes (cc)		162.8	491.0	118.0	219.0	307.8
COIN of prostate	DMPO	1.40	3.28	8.51	9.82	16.9
	DAO	1.83	6.41	8.76	10.7	15.9
COIN of nodes	DMPO	1.96	1.93	2.83	2.79	1.95
	DAO	2.42	1.59	2.97	3.09	1.86

### B. Analysis of segment information

**Figure 1:**
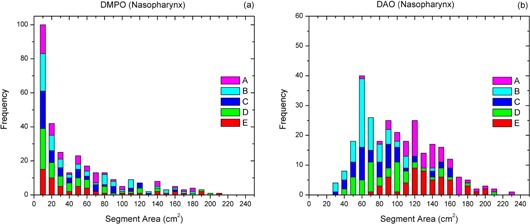
Frequency analysis of segment area information of (a) DMPO and (b) DAO plans for five patients (A–E) with nasopharyngeal cancer.

**Figure 2:**
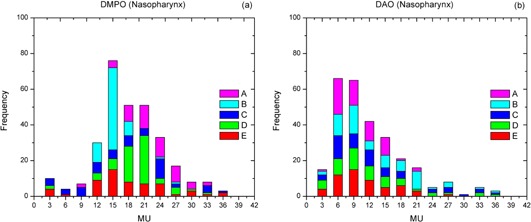
Frequency analysis of segment MU information of (a) DMPO and (b) DAO plans for five patients (A–E) with nasopharyngeal cancer.

The distributions of SAs and MUs of five DMPO and DAO plans were calculated for five patients (A–E) with nasopharyngeal cancer. For the DMPO plans, SAs of $2-20\;{\text{cm}}^{2}$ were predominantly used (see Fig. 1(a)). For the DAO plans, SAs ranging from 40 to 160 cm^2^ were more frequently used (see Fig. 1(b)). The most frequently occurring MUs per segment were in the range of 9–24 and 3–15 (see Figs. 2(a) and 2(b)) for the DMPO and DAO plans, respectively.

**Figure 3:**
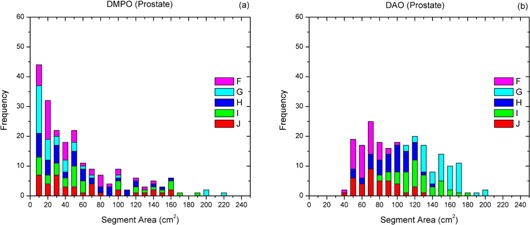
Frequency analysis of segment area information of (a) DMPO and (b) DAO plans for five patients (F–J) with prostate cancer.

**Figure 4:**
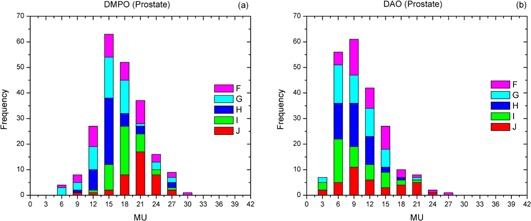
Frequency analysis of segment MU information of (a) DMPO and (b) DAO plans for five patients (F–J) with prostate cancer.

Similar to the nasopharynx cases, the DMPO plans for five patients (F–J) with advanced prostate cancer consisted of more small area segments with large segmental MUs compared to the DAO plans. For the DMPO plans, the most frequently employed SAs and MUs per segment were from 2 to 50 cm^2^ (see Fig. 3(a)) and from 6 to 24 (see Fig. 4(a)), respectively. For the DAO plans, the most frequently used SAs and MUs per segment ranged from 40 to 170 cm^2^ (see Fig. 3(b)) and from 3 to 15 (see Fig. 4(b)), respectively. It was noticed that the DMPO plan for patient G had many segments larger than 180 cm^2^, which may be caused by the large CTV (see Table 2). The average SA (85.3 cm^2^) for this DMPO plan, however, was still smaller than that for the corresponding DAO plan (145.7 cm^2^).

### C. Effects of DMPO optimization parameters on efficiency

For both nasopharynx and prostate cases, the use of 16 cm^2^ MSA was about the limit for DMPO to generate a comparable plan as the clinically approved plan.

**Figure 5:**
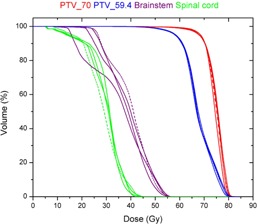
DVHs of four DMPO plans for a patient with nasopharyngeal cancer. These plans were created with the same treatment goals and optimization settings except for the MSA settings, which were 2 (red), 4 (blue), 8 (green), and 16 cm^2^ (magenta), respectively. The brain stem was plotted with dashed lines to distinguish from the spinal cord.

For a nasopharynx case, the DVHs of selected target volumes and OARs of four DMPO plans, with the MSA set to 2, 4, 8, and 16 cm^2^, are shown in Fig. 5. Compared to the clinically approved plan $\left(\text{MSA}=2\;{\text{cm}}^{2}\right)$, the DVHs of the PTV70, and the PTV59.4 were in agreement for the other three DMPO plans. Although the DVHs of the spinal cord and brain stem were different in the low‐dose range, the dosimetric constraints for these and other OARs (based on RTOG HN‐0225) were still met (see Table 1).

**Figure 6:**
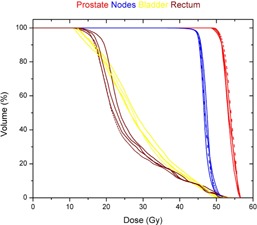
DVHs of four DMPO plans for a patient treated with prostate and lymph nodes. These plans were created with the same treatment goals and optimization settings except for the MSA settings, which were 2 (red), 4 (blue), 8 (green), and 16 cm^2^ (magenta), respectively. The bladder was plotted with dashed lines to distinguish from the rectum.

For a prostate case, the resulting DVHs of four DMPO plans, with the MSA set to 2, 4, 8, and 16 cm^2^, are shown in Fig. 6. The dosimetric endpoints to the prostate, pelvic lymph nodes, rectum, and bladder were comparable among all DMPO plans.

**Figure 7:**
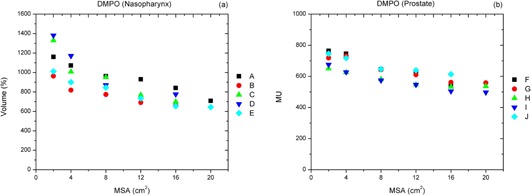
Dependence of plan monitor units (MUs) on the MSA setting for DMPO plans created for (a) five patients (A–E) with nasopharyngeal cancer, and (b) five patients (F–J) with prostate cancer. The clinical plans were created with $\text{MSA}=2\;{\text{cm}}^{2}$, and other plans were created with MSA equal to 4, 8, 12, 16, and 20 cm^2^ (3 patients only), respectively.

For five nasopharynx and five prostate cases, the DMPO plans with the MSA of 16 cm^2^ required 38% (727 vs. 1,169) and 22% (552 vs. 711) less MUs compared to the clinically approved DMPO plans. As seen in Fig. 7, the number of MUs for the DMPO plans decrease as the MSA setting increases, but the reduction of MUs tends to decrease when the MSA approaches $12-16\;{\text{cm}}^{2}$ or larger.

## IV. DISCUSSION

The complexity of IMRT plans, measured by the total number of MUs and the number of segments, substantially impacts many aspects of IMRT treatment: the possibility of radiation‐induced cancers,^2^, ^3^, ^23^, ^24^ the treatment time,^(25)^ and the quality and accuracy of radiotherapy.^25^, ^26^ Much effort, mostly on the development of new optimization algorithms, has been devoted to reducing the complexity of IMRT plans. By directly incorporating delivery constraints into optimization, aperture‐based (or one‐step) optimization has been proved as a means of achieving improved MU efficiency over conventional two‐step optimization.^4^, ^5^, ^6^, ^7^, ^8^, ^9^, ^10^, ^11^, ^12^, ^13^, ^14^, ^15^, ^16^ Broderick et al.^(16)^ summarized different aperture‐based optimization algorithms in an excellent review article. Further effort^20^, ^26^ has been devoted to finding the minimum number of segments in order to reduce the complexity of IMRT plans and to decrease the total MU required. In our practice, we observed that the total number of segments is not the only indicator for MU efficiency. With a given number of segments, the distributions and selections of large area segments also played an important role in MU reduction.

In this paper, we investigated the relationship between SA and MU efficiency by comparing IMRT plans created using two aperture‐based optimization algorithms, DMPO and DAO, implemented in two commercially available treatment planning systems. While achieving a similar plan quality as DMPO, DAO saved an average of 43% (from 1,169 to 672) and 44% MUs (from 711 to 400) for five nasopharynx and five prostate cases, respectively. Further analysis of the relationship between SAs and MUs of each plan indicated that the DMPO plans tended to use more small area segments than the DAO plans. Specifically, the DMPO plans consisted of a large number of segments with SA close to the MSA setting of 2 cm^2^, and the DAO plans had more segments with SA ranging from 40 to 160 cm^2^, which was substantially greater than the MSA setting of 2 cm^2^. In step‐and‐shoot IMRT plans, the relationship between MSA and plan quality was not well understood. In practice, planners often set the MSA to a small value (e.g., 2 cm^2^) to ensure a clinically acceptable IMRT can be generated. An interesting observation in this study was that both DMPO (if MSA was set to a large value) and DAO could result in clinically acceptable IMRT plans by using relatively large‐sized segments.

One of the limitations of this study was that two treatment planning systems utilized different dose calculation engines. In one of our previous studies,^(27)^ we compared the differences between two dose calculations by inputting DAO plans into the Pinnacle system. After recalculating the DAO plans with the same MUs and shapes of segments, we found no significant differences in the two dose calculation methods. Another limitation was that we did not investigate the optimal setting of the minimum segment area (MSA) parameter for each clinical site. However, for some cases in this study, we explored a series of DMPO plans with increased values of the MSA, such as 2, 4, 8, and 16 cm^2^ (the MSA of 2 cm^2^ for the clinical plans). With comparable plan qualities in these DMPO plans, the MUs were reduced 38% (from 1,169 to 727) for the nasopharynx cases and 23% (from 711 to 551) for the prostate cases when the MSA increased from 2 to 16 cm^2^. Similar results were observed by Worthy and Wu^(28)^ in their study of DMPO for head‐and‐neck cancer. With plans delivered by Elekta linear accelerators (Elekta, Norcross, GA), they suggested the MSA should be set to 8 cm^2^ or larger to avoid the local minimum and to achieve improved delivery efficiency. We also observed that the extent of MU reduction reached a plateau as the MSA reached $12-16\;{\text{cm}}^{2}$ (see Fig. 5). Future study is required to confirm this observation.

Craft et al.^(29)^ investigated the tradeoff between IMRT complexity (measured as the number of MUs) and plan quality. They concluded that the largely increased number of MU and irradiation time in IMRT is sometimes unnecessary, and they suggested a constraint upon plan MUs. Our study, in part, supported this suggestion. Based on the results of this paper, we would suggest that a new constraint can be applied upon MSA for DMPO to allow only a certain number of segments to have SA close to the MSA setting.

Although DMPO and DAO started with the same MSA, the resultant distributions of segments used for step‐and‐shoot IMRT plans were drastically different in terms of SA and MUs per segment. The difference may stem from the optimization search method implemented in the Pinnacle and Panther systems. The Pinnacle TPS uses a gradient search method for DMPO, while the Panther TPS uses a simulated annealing method for DAO. In general, aperture‐based optimization is likely to be trapped in a local minimum if a gradient search method is employed. To reduce this possibility, the Pinnacle TPS divides the DMPO into two parts. In the first part of optimization, a continuous fluence optimization is performed so that the solution found in this part of optimization is near optimal although the delivery parameters are not considered. After considering delivery parameters, the solution found in the first part of optimization will be deteriorated. Therefore, the second part of the optimization is to remedy the deteriorated plan by further adjusting the MLC positions and segment weights without increasing the number of segments. The starting point for the second optimization tends to include many segments with SA close to the MSA setting, generally as small as $2-4\;{\text{cm}}^{2}$. During this 2nd optimization, the gradient search method in the Pinnacle system cannot generate solutions consisting of segments substantially different from those initial segments, and this might be the reason that MUs from DMPO plans were higher than those from DAO plans.

The result of this study may help us to understand the improvement of MU efficiency in the recently emerging volumetric‐modulated arc therapy (VMAT) technique. It has been reported that aperture‐based optimization is employed in VMAT plans with many more beam angles arranged in single or double arcs. For example, Otto^(30)^ used a similar strategy as the DAO approach to incorporated MLC leaf positions and MU weights into his VMAT optimization. Most reported MU reductions of the VMAT plans were comparable to the sliding‐window IMRT plans.^31^, ^32^ According to our findings in this study, depending on the implementation of aperture‐based optimization, MU saving in DAO plans is comparable to those reported for VMAT plans.^31^, ^32^

## V. CONCLUSIONS

While achieving comparable plan quality with similar optimization parameters, DMPO and DAO can result in large differences in MU efficiency for step‐and‐shoot IMRT plans. The results of this study have shown that improved MU efficiency is also dependent on the ability of an optimization algorithm and the setting of the optimization parameters to create IMRT plans with large area segments.

## ACKNOWLEDGMENTS

This research is supported in part by the United States Army Medical Research and Material Command (USAM/RMC, PC073349) and in part by a research grant from Siemens Medical Solutions.

## References

[1] Ludlum E and Xia P . Comparison of IMRT planning with two‐step and one‐step optimization: a way to simplify IMRT. Phys Med Biol. 2008;53(3):807–21. 18199916 10.1088/0031-9155/53/3/018

[2] Hall EJ and Wuu CS . Radiation‐induced second cancers: the impact of 3D‐CRT and IMRT. Int J Radiat Oncol Biol Phys. 2003;56(1):83–88. 12694826 10.1016/s0360-3016(03)00073-7

[3] Kry SF , Salehpour M , Followill DS , et al. The calculated risk of fatal secondary malignancies from intensity‐modulated radiation therapy. Int J Radiat Oncol Biol Phys. 2005;62(4):1195–203. 15990025 10.1016/j.ijrobp.2005.03.053

[4] De Gersem W , Claus F , De Wagter C , De Neve W . An anatomy‐based beam segmentation tool for intensity‐modulated radiation therapy and its application to head‐and‐neck cancer. Int J Radiat Oncol Biol Phys. 2001;51(3):849–59. 11699497 10.1016/s0360-3016(01)01727-8

[5] Shepard DM , Earl MA , Li XA , Naqvi S , Yu C . Direct aperture optimization: a turnkey solution for step‐and‐shoot IMRT. Med Phys. 2002;29(6):1007–18. 12094970 10.1118/1.1477415

[6] Siebers JV , Lauterbach M , Keall PJ , Mohan R . Incorporating multi‐leaf collimator leaf sequencing into iterative IMRT optimization. Med Phys. 2002;29(6):952–59. 12094990 10.1118/1.1477230

[7] Cotrutz C and Xing L . Segment‐based dose optimization using a genetic algorithm. Phys Med Biol. 2003;48(18):2987–98. 14529206 10.1088/0031-9155/48/18/303

[8] Y. Li , Yao J , Yao D . Genetic algorithm based deliverable segments optimization for static intensity‐modulated radiotherapy. Phys Med Biol. 2003;48(20):3353–74. 14620063 10.1088/0031-9155/48/20/007

[9] Bedford JL and Webb S . Constrained segment shapes in direct‐aperture optimization for step‐and‐shoot IMRT. Med Phys. 2006;33(4):944–58. 16696471 10.1118/1.2163832

[10] Bergman AM , Bush K , Milette MP , Popescu IA , Otto K , Duzenli C . Direct aperture optimization for IMRT using Monte Carlo generated beamlets. Med Phys. 2006;33(10):3666–79. 17089832 10.1118/1.2336509

[11] Bedford JL and Webb S . Direct‐aperture optimization applied to selection of beam orientations in intensity‐modulated radiation therapy. Phys Med Biol. 2007;52(2):479–98. 17202628 10.1088/0031-9155/52/2/012

[12] Mestrovic A , Milette MP , Nichol A , Clark BG , Otto K . Direct aperture optimization for online adaptive radiation therapy. Med Phys. 2007;34(5):1631–46. 17555245 10.1118/1.2719364

[13] Jones S and Williams M . Clinical evaluation of direct aperture optimization when applied to head‐and‐neck IMRT. Med Dosim. 2008;33(1):86–92. 18262129 10.1016/j.meddos.2007.04.002

[14] van Asselen B , Schwarz M , van Vliet‐Vroegindeweij C , Lebesque J V , Mijnheer BJ , Damen EM . Intensity‐modulated radiotherapy of breast cancer using direct aperture optimization. Radiother Oncol. 2006;79(2):162–69. 16712992 10.1016/j.radonc.2006.04.010

[15] Ahunbay EE , Chen GP , Thatcher S , et al. Direct aperture optimization‐based intensity‐modulated radiotherapy for whole breast irradiation. Int J Radiat Oncol Biol Phys. 2007;67(4):1248–58. 17275205 10.1016/j.ijrobp.2006.11.036

[16] Broderick M , Leech M , Coffey M . Direct aperture optimization as a means of reducing the complexity of Intensity Modulated Radiation Therapy plans. Radiat Oncol. 2009;4:8. 19220906 10.1186/1748-717X-4-8 PMC2647925

[17] Lee N , Harris J , Garden AS , et al. Intensity‐modulated radiation therapy with or without chemotherapy for nasopharyngeal carcinoma: radiation therapy oncology group phase II trial 0225. J Clin Oncol. 2009;27(22):3684–90. 19564532 10.1200/JCO.2008.19.9109 PMC2720082

[18] Xia P , Lee N , Liu YM , et al. A study of planning dose constraints for treatment of nasopharyngeal carcinoma using a commercial inverse treatment planning system. Int J Radiat Oncol Biol Phys. 2004;59(3):886–96. 15183492 10.1016/j.ijrobp.2004.02.040

[19] Chan LW , Xia P , Gottschalk AR , et al. Proposed rectal dose constraints for patients undergoing definitive whole pelvic radiotherapy for clinically localized prostate cancer. Int J Radiat Oncol Biol Phys. 2008;72(1):69–77. 18342454 10.1016/j.ijrobp.2007.12.045

[20] Jiang Z , Earl MA , Zhang GW , Yu CX , Shepard DM . An examination of the number of required apertures for step‐and‐shoot IMRT. Phys Med Biol. 2005;50(23):5653–63. 16306659 10.1088/0031-9155/50/23/017

[21] Mackie TR , Scrimger JW , Battista JJ . A convolution method of calculating dose for 15‐MV x rays. Med Phys. 1985;12(2):188–96. 4000075 10.1118/1.595774

[22] Papanikolaou N , Mackie TR , Meger‐Wells C , Gehring M , Reckwerdt P . Investigation of the convolution method for polyenergetic spectra. Med Phys. 1993;20(5):1327–36. 8289713 10.1118/1.597154

[23] Shaw E , Scott C , Souhami L , et al. Single dose radiosurgical treatment of recurrent previously irradiated primary brain tumors and brain metastases: final report of RTOG protocol 90–05. Int J Radiat Oncol Biol Phys. 2000;47(2):291–98. 10802351 10.1016/s0360-3016(99)00507-6

[24] Feuvret L , Noel G , Mazeron JJ , Bey P . Conformity index: a review. Int J Radiat Oncol Biol Phys. 2006;64(2):333–42. 16414369 10.1016/j.ijrobp.2005.09.028

[25] Galvin JM , Ezzell G , Eisbrauch A , et al. Implementing IMRT in clinical practice: a joint document of the American Society for Therapeutic Radiology and Oncology and the American Association of Physicists in Medicine. Int J Radiat Oncol Biol Phys. 2004;58(5):1616–34. 15050343 10.1016/j.ijrobp.2003.12.008

[26] Lee MT , Purdie TG , Eccles CL , Sharpe MB , Dawson LA . Comparison of simple and complex liver intensity modulated radiotherapy. Radiat Oncol. 2010;5:115. 21114865 10.1186/1748-717X-5-115 PMC3003186

[27] Xia P , Qi P , Rembert J , Hu AZ , Quivey JM , Yom SS . A treatment planning method to avoid the larynx and eliminate the match‐line in the treatment of head and neck cancer with intensity‐modulated radiation therapy. J Radiat Oncol. 2012;1(2):187–94.

[28] Worthy D and Wu Q . Parameter optimization in HN‐IMRT for Elekta linacs. J Appl Clin Med Phys. 2009;10(2):2951. 19458598 10.1120/jacmp.v10i2.2951 PMC5720449

[29] Craft D , Süss P , Bortfeld T . The tradeoff between treatment plan quality and required number of monitor units in intensity‐modulated radiotherapy. Int J Radiat Oncol Biol Phys. 2007;67(5):1596–605. 17394954 10.1016/j.ijrobp.2006.11.034

[30] Otto K . Volumetric modulated arc therapy: IMRT in a single gantry arc. Med Phys. 2008;35(1):310–17. 18293586 10.1118/1.2818738

[31] Wiezorek T , Brachwitz T , Georg D , et al. Rotational IMRT techniques compared to fixed gantry IMRT and tomotherapy: multi‐institutional planning study for head‐and‐neck cases. Radiat Oncol. 2011;6:20. 21338501 10.1186/1748-717X-6-20 PMC3050734

[32] Yoo S , Wu QJ , Lee WR , Yin FF . Radiotherapy treatment plans with RapidArc for prostate cancer involving seminal vesicles and lymph nodes. Int J Radiat Oncol Biol Phys. 2010;76(3):935–42. 20044214 10.1016/j.ijrobp.2009.07.1677

